# Visual attention for social information and salivary oxytocin levels in preschool children with autism spectrum disorders: an eye-tracking study

**DOI:** 10.3389/fnins.2014.00295

**Published:** 2014-09-17

**Authors:** Takashi X. Fujisawa, Shiho Tanaka, Daisuke N. Saito, Hirotaka Kosaka, Akemi Tomoda

**Affiliations:** ^1^Research Center for Child Mental Development, University of FukuiFukui, Japan; ^2^Department of Child Development, United Graduate School of Child Development, Osaka University, Kanazawa University, Hamamatsu University School of Medicine, Chiba University, University of FukuiFukui, Japan; ^3^Biomedical Imaging Research Center, University of FukuiFukui, Japan; ^4^Department of Neuropsychiatry, Faculty of Medical Sciences, University of FukuiFukui, Japan

**Keywords:** oxytocin (OT), autism spectrum disorder (ASD), visual attention, preschool children, eye-tracking

## Abstract

This study was designed to ascertain the relationship between visual attention for social information and oxytocin (OT) levels in Japanese preschool children with autism spectrum disorder (ASD). We hypothesized that poor visual attention for social information and low OT levels are crucially important risk factors associated with ASD. We measured the pattern of gaze fixation for social information using an eye-tracking system, and salivary OT levels by the Enzyme-Linked Immunosorbent Assay (ELISA). There was a positive association between salivary OT levels and fixation duration for an indicated object area in a finger-pointing movie in typically developing (TD) children. However, no association was found between these variables in children with ASD. Moreover, age decreased an individual's attention to people moving and pointed-at objects, but increased attention for mouth-in-the-face recognition, geometric patterns, and biological motions. Thus, OT levels likely vary during visual attention for social information between TD children and those with ASD. Further, aging in preschool children has considerable effect on visual attention for social information.

## Introduction

Oxytocin (OT), a neuropeptide secreted from the posterior pituitary, has physiological functions in labor and lactation, and there is increasing evidence that OT plays an important role in modulating social behavior in diverse species (Donaldson and Young, 2008; Insel, 2010). In humans, much research has suggested that OT facilitates the ability to infer the mental state of others from the eye region (i.e., Domes et al., 2007b; Guastella et al., 2008, 2010), and can even selectively enhance the memory encoding of faces (Rimmele et al., 2009). OT also modulates trust and generosity in interpersonal relationships (Kosfeld et al., 2005; Zak et al., 2005, 2007; Baumgartner et al., 2008; Kéri et al., 2009). In fact, OT affects the activation of brain areas responsible for emotion, mentalization, and cognitive control, including the amygdala and prefrontal cortex (Kirsch et al., 2005; Domes et al., 2007a; Baumgartner et al., 2008). In addition, oxytocin receptors (OXTR) are expressed in brain areas, such as medial prefrontal cortex (MPFC), dorsal anterior cingulate cortex (dACC), amygdala and dorsal striatum (Landgraf and Neumann, 2004; Skuse and Gallagher, 2009), that are involved in social behavior, including reproductive and maternal behaviors, affiliation and attachment, and reactivity to social stress in nonhuman mammals (Carter, 1998; Ferguson et al., 2000; Young and Wang, 2004). Further, OXTR gene polymorphisms were associated with prosocial behavior in a dictator game and in social value orientations tasks (Israel et al., 2009).

Autism spectrum disorder (ASD) is a common neurodevelopmental disorder affecting around 3.8/1000 boys and 0.8/1000 girls (Taylor et al., 2013). Revised diagnostic criteria for ASDs recently published in the latest Diagnostic and Statistical Manual of Mental Disorders (DSM-5), include two core areas: communication and social deficits, and fixed or repetitive behaviors (American Psychiatric Association, 2013). A core symptom of ASD is indeed the presence of early and persistent deficits in social interaction and social communication. Individuals with ASD experience difficulties in establishing and maintaining eye contact and in processing facial information and intentions (American Psychiatric Association, 2013). Individuals with ASD have particular difficulty in interpreting nonverbal social information such as facial expressions, vocal expressions (Doi et al., 2013), pointing gestures (Paparella et al., 2011), and body gestures (Centelles et al., 2012) that lead to severe social impairment (Hill and Frith, 2003). Recently, many studies have revealed relations between OT and social functioning (e.g., Munesue et al., 2010; Higashida et al., 2012), and have devoted particular attention to OT as a candidate treatment for social impairments in patients with ASD. In fact, plasma OT levels in patients with ASDs are reportedly low (Modahl et al., 1998). Moreover, nasal administration of OT improve social impairments in patients with ASDs (Andari et al., 2010; Guastella et al., 2010; Kosaka et al., 2012; Watanabe et al., 2014).

Several studies have revealed that patients with ASD have impaired visual attention for social information when compared to individuals that have undergone typical development (e.g., Klin et al., 2002b, 2009; Nakano et al., 2010; Pierce et al., 2011; Jones and Klin, 2013; Sasson and Touchstone, 2014). In that context, eye-tracking technology can provide great benefits for investigating visual attention regarding social information in ASD. This approach enables researchers to measure, with high precision and accuracy, what a participant is looking at and for how long. Moreover, it offers an optimal balance between ecological validity and methodological constraints (Guillon et al., 2014). Eye tracking is therefore a unique method to detect and characterize subtle variations in the spontaneous viewing patterns of individuals with ASD (Klin et al., 2002a, 2003). Moreover, eye-tracking technology is applicable for all populations, from infants to adults, irrespective of their level of non-verbal and verbal ability (Guillon et al., 2014). Therefore, the different aspects of visual attention for social information can be investigated similarly across various participant statuses, such as age, sex, and clinical condition.

The aim of this study was to investigate the relationship between visual attention for social information and salivary OT levels in preschool children with ASD compared to children with typical development. In terms of peripheral OT, studies have reported correlations between plasma and salivary OT concentrations in humans (Carter et al., 2007; Feldman et al., 2011). Although several studies have examined the relationship between social dysfunction and peripheral OT levels, or between social dysfunction and the pattern of visual attention using eye-tracking, the association between the two in preschool children with ASD and typical development remains unclear. Moreover, developmental patterns change with age, and sex differences could exist; thus, it is surprising that these aspects of ASD have not been investigated. Here, we measured the pattern of visual attention for social information and salivary OT levels using eye-tracking and Enzyme-Linked Immunosorbent Assay (ELISA), respectively, to investigate the relationship among these factors. We hypothesized that poor visual attention for social information and low salivary OT levels are crucially important risk factors associated with ASD.

## Materials and methods

### Ethics statement

The Ethics Committee of the University of Fukui approved the study protocol (Assurance # FU24-123), and the parents of all participants gave written informed consent. The experimental protocol was conducted in accordance with the Declaration of Helsinki.

### Participants

In this study, 19 preschool children with ASD (16 boys and 3 girls, mean age, 57.9 months; *SD*, 13.6 months) participated along with 60 typically developing (TD) preschoolers (28 boys and 32 girls, mean age, 48.1 months; *SD*, 22.7 months) (Table 1). Subjects in the ASD group were predominantly male (84%), whereas TD controls were skewed slightly female (53%). Six children (4 with ASD, 2 TD) were excluded from our final analyses because of noncompliance during testing, which was attributable to fewer than 50% of valid trials in the eye-tracking task or to insufficient saliva samples.

**Table 1:** **Characteristics of subjects used in the dataset for statistical analysis**.

**Characteristic**	**TD**	**ASD**	***p*-value**
Subjects numbers (male/female)	60 (28/32)	19 (16/3)	<0.05 (χ^2^-test)
Age (month)	48.1 (22.7)	57.9 (13.6)	*n.s*. (*t*-test)
DQ (Kyoto scale)	–	77.7 (19.5)	–
PARS score (peak)	–	20.9 (8.50)	–
PARS score (current)	–	18.6 (8.68)	–
SDQ score	–	17.7 (4.36)	–

*TD, Typical Development; ASD, Autism spectrum disorder; DQ, Developmental Quotient; PARS, Pervasive Developmental Disorders Autism Society Japan Rating Scale; SDQ, Strength and Difficulties Questionnaire*.

The patients were referred to our laboratory during 2013–2014 for examination of visual attention and OT measurement in saliva. All patients were referred from the Department of Child and Adolescent Psychological Medicine, University of Fukui Hospital. All participants' race/ethnicity was Japanese. Diagnoses were made by one senior pediatric neurologist through interviews and reviews of clinical records, according to the DSM-5 (American Psychiatric Association, 2013). We assessed their developmental status using the developmental quotient (DQ) (Kyoto Scale of Psychological Development), PARS (Pervasive Developmental Disorders Autism Society Japan Rating Scale), and SDQ (Strength and Difficulties Questionnaire). The validity and reliability of the Japanese version of these scales were confirmed (Ikuzawa et al., 2002; Tsujii et al., 2006; Matsuishi et al., 2008; Iizuka et al., 2010). The detailed characteristics of the participants included in the final analysis are summarized in Table 1.

To obtain data from normal age-matched typically developed controls, healthy preschool children were recruited as subjects from the community. Kindergarten students were targeted, and none had any physical problem or had encountered any abnormal developmental milestone.

### Measures of OT levels in saliva

Saliva samples were collected using Salivettes® (Sarstedt, Rommelsdorft, Germany). Parents were asked to put a roll of cotton in their child's mouth and instructed their child to chew for 1 min until it was saturated with saliva. Two cotton samples were collected by repeating the chewing process twice. Saliva samples were frozen and stored at −80°C in the laboratory. Before the assay was run, saliva samples were lyophilized overnight and kept at −20°C to concentrate them 2–4 times. The dry samples were reconstructed in the assay buffer immediately before analysis using an OT enzyme immunoassay commercial kit (Assay Designs Inc., Ann Arbor, MI). These protocols were consistent with an earlier study for adults (Carter et al., 2007; White-Traut et al., 2009; Feldman et al., 2011; Gordon et al., 2013), as well as one that had been performed on children at the age of 3 years old (Feldman et al., 2013). Each sample was performed in duplicate and concentrations were calculated using the SpectraMax® (Molecular Device, Sunnyvale, California) micro plate reader, according to relevant standard curves. Average intra- and inter-assay coefficients of variation (CV) were 5.7 and 11.7%, respectively.

### Measures of the gaze pattern

**Figure 1:**
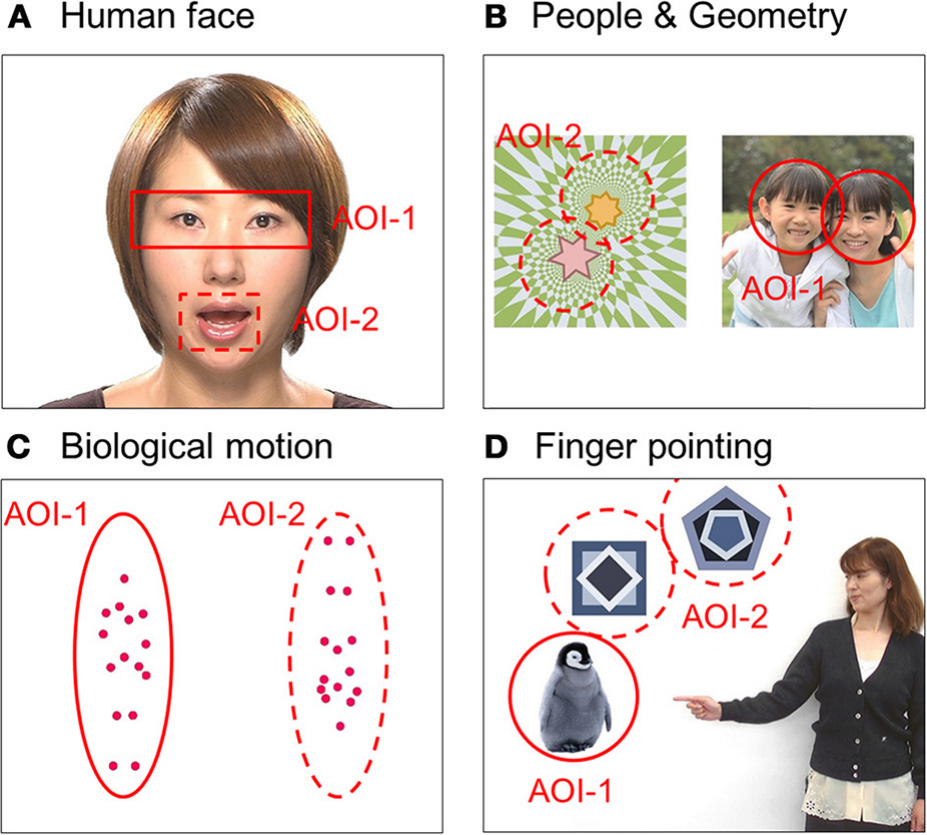
**Snapshots of four short movies with each type of social information as stimuli presented by the Gazefinder®: (A) human faces, (B) people and geometric patterns, (C) bodily motions of a human, and (D) objects with or without finger pointing**. These stimuli were set as two areas-of-interest (AOI-1 and AOI-2).

We measured each child's gaze pattern using Gazefinder® (JVC Kenwood; Hamamatsu, Japan) for response to social information by visual stimuli. The Gazefinder® used infrared light sources and cameras that were integrated into a 19-inch-thin film transistor monitor (1280 × 1024 pixels). Using corneal reflection techniques, the Gazefinder® records the X and Y coordinates of each child's eye position at a frequency of 50 Hz (i.e., 3000 data collections/min). Stimuli presented by the Gazefinder® consisted of short movies including four categories of social information, which were (A) human faces, (B) people and geometric patterns, (C) bodily motion of a human, and (D) objects with or without finger pointing. These stimuli were set to two areas as areas-of-interest (AOI), which included the eyes and mouth areas in the human face category (31 s), people and geometric image areas in the people and geometric patterns category (32 s), upright and inverted image areas in the motion category (20 s), and lastly, an object with or without pointing (20 s) (Figure 1).

### Procedure

Testing was executed in a research laboratory located in the Research Center for Child Mental Development at University of Fukui. Children were seated (on a parent's lap when younger than 18 months) 40 cm in front of the eye-tracking monitor. To obtain calibration information, children were first shown images of an animated animal that appeared in one of five locations on the screen. If the calibration quality was poor for any of these points, then the calibration process was repeated. Before the task, children were told that pictures of faces, people, and objects were to be shown on the computer screen and that they should look at them without looking away for as long as possible. Stimulus movies were displayed in a definitive order. Before each trial, an attention-getting animation with a voice saying “Hey! Look!” was presented in the center of the screen to reorient their attention for stimuli.

### Statistical procedure

We computed the percentage of fixation durations on two AOIs as well as for other areas on the screen, and analyzed them as dependent variables of social attention relevant to the autistic phenotype. Analysis of visual attention for social information between the groups was then conducted using a separate repeated measures analysis of variance (ANOVA) for each primary variable, with a cue type (face, people, motion, and pointing) as the within-subjects variable and group (ASD, TD) as the between group variable. The significance level was set to *p* < 0.05. Statistical analyses were conducted using software (IBM SPSS 20.0 for Windows, Statistical Package for the Social Sciences; IBM).

## Results

### Typical development of visual attention for social information and salivary OT levels

Mean values of salivary OT levels and the mean percentage of fixation durations for each category of social information in TD children are presented in Table 2, together with standard errors. Mean values of these variables by sex are also shown. For salivary OT levels and the percentage of fixation durations, we tested the effect of sex difference separately for each variable. First, for the salivary OT levels, no significant difference was observed [*t*_(56)_ = 0.35, *n.s*.], indicating that no sex difference of OT levels existed in either group. Next, for the face stimuli, a significant difference was observed in the % of fixation on the mouth [*t*_(56)_ = 1.84, *p* < 0.10], indicating that female TD children were more attentive to the mouth area in faces than male children, although no sex-related difference of attention for the eye area was found between groups [*t*_(56)_ = 0.93, *n.s*.]. For the people and geometry stimuli, a significant difference was observed in the % of fixation on people stimuli [*t*_(56)_ = 3.42, *p* < 0.001], indicating that female TD children were more attentive to people moving than male children; however, no sex-related difference in attention for geometry patterns was found between groups [*t*_(56)_ = 1.52, *n.s*.]. For bodily motion stimuli, a significant difference was observed in the fixation % on stimuli of upright positions [*t*_(56)_ = 3.10, *p* < 0.01], indicating that female TD children were more attentive to biological motion than were male children, although no sex-related difference in attention for inverted presentation of bodily motion was found between groups [*t*_(56)_ = 0.38, *n.s*.]. Finally, for finger-pointing stimuli, a significant difference was observed in the % of fixation on pointed objects [*t*_(56)_ = 2.38, *p* < 0.05], indicating that female TD children were more attentive to pointed-at objects than male children; however, no sex difference of attention for non-pointed-at objects was found between groups [*t*_(56)_ = 0.66, *n.s*.].

**Table 2:** **Mean values and correlation coefficients of salivary OT levels and gaze fixation parameters in TD children**.

		**Total (***n* = 58**)**		**Male (***n* = 27**)**		**Female (***n* = 31**)**		***t***	**Correlation**		**Partial correlation**
		**Mean**	***SD***	**Mean**	***SD***	**Mean**	***SD***		**Age**	**OT level**	**OT level**
Oxytocin levels (pg/ml)		44.5	24.89	45.7	29.78	43.4	20.15	0.35	−0.164	–	–
Face	%Eyes	39.8	13.78	40.1	14.78	39.6	13.09	0.13	−0.090	−0.011	−0.028
	%Mouth	34.4	13.73	**30.9**	13.48	**37.5**	13.42	**1.84^#^**	0.401^**^	−0.106	−0.028
	%Out of AOI	13.4	6.86	14.3	7.89	12.7	5.83	0.93	−0.087	−0.063	−0.087
People and geometry	%People	45.0	15.21	**38.3**	14.95	**50.8**	13.03	3.42^***^	−0.466^**^	0.007	−0.059
	%Geometry	29.1	16.54	32.6	19.34	26.0	13.22	1.52	0.576^**^	−0.012	0.093
	%Out of AOI	18.0	6.27	18.1	6.02	18.0	6.59	0.06	−0.061	−0.020	−0.032
Biological motion	%Upright	51.6	14.98	**45.6**	14.48	**56.9**	13.50	3.10^**^	0.315^*^	0.016	0.109
	%Inverted	34.5	13.07	35.2	12.93	33.9	13.38	0.38	0.042	−0.081	−0.078
	%Out of AOI	8.0	7.65	9.6	10.01	6.6	4.46	1.44	−0.154	−0.009	−0.049
Finger pointing	%Pointed	43.0	12.19	**39.1**	12.71	**46.5**	10.80	2.38^*^	−0.352^**^	0.281^*^	0.273^*^
	%Non-pointed	10.7	6.77	10.0	6.83	11.2	6.78	0.66	0.441^**^	−0.145	−0.075
	%Out of AOI	38.6	10.27	38.2	9.93	38.9	10.70	0.26	0.220	−0.040	−0.001

# p < 0.10;

* p < 0.05;

** p < 0.01;

*** *p < 0.001*.

**Figure 2:**
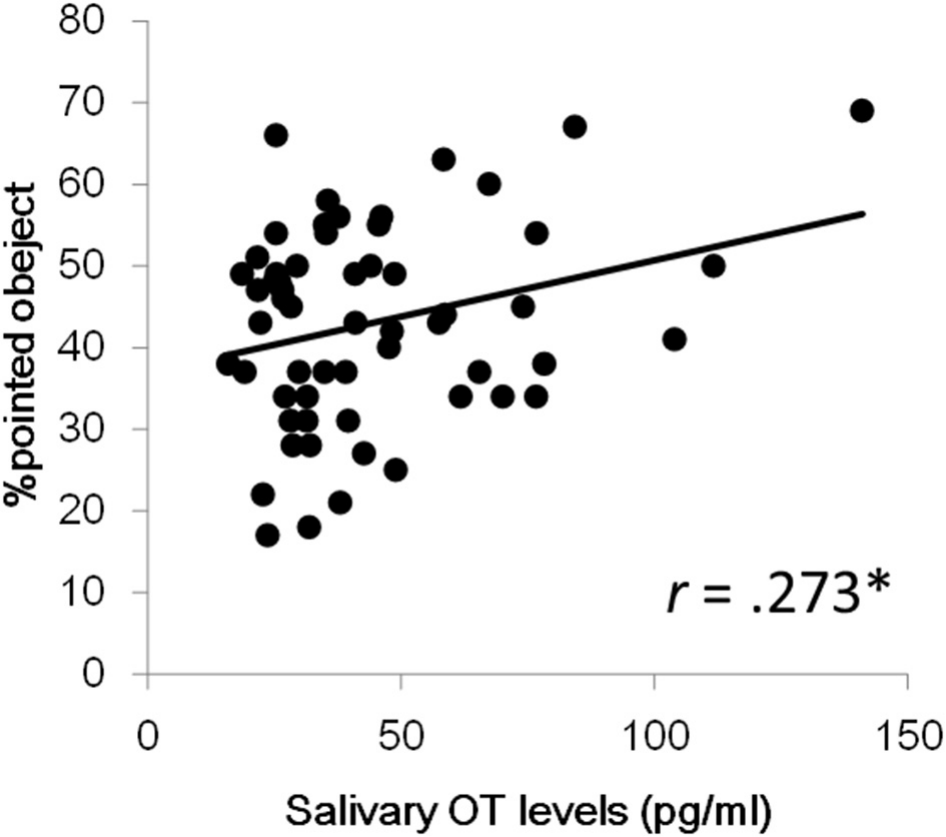
**Relationship between salivary OT levels and % of pointed-at objects in the finger pointing movies**. ^*^p < 0.05.

Next, we investigated aging effects for salivary OT levels and fixation durations for each social measure in order to address whether the pattern of attention for social information is dependent on age. We also wanted to address this since the relationship between OT levels and aging in infant and preschool children remains unclear. The correlation coefficients between age, salivary OT levels, and each variable of fixation duration are also presented in Table 2. First, no significant correlation was found between age and OT levels. Next, for fixation durations, a significant positive correlation was observed for mouth area of the facial stimuli, geometric pattern stimuli, and upright body position. Significant negative correlations were found for stimuli showing people moving and pointed-at objects in finger-pointing stimuli. These results suggest that aging reduces attention for human movement and for pointed-at objects, but that aging increases a TD child's attention for mouths, geometric patterns, and biological motion. Finally, a significant correlation of salivary OT levels was found only with the fixation % of pointed-at objects in finger-pointing stimuli. To exclude the effects of sex and age, we calculated partial correlation coefficients between salivary OT levels and the % of pointed-at objects, controlled by sex and age. Results showed significant partial correlation coefficients between the variables, indicating that children with high OT levels were more attentive to the pointed-at object than children with low OT levels (Figure 2).

### Development of visual attention for social signals and OT levels with ASD

Together with standard errors, mean values of salivary OT and proportions of gaze fixations for each category of social information in children with ASD are presented in Table 3. Mean values of these parameters grouped by sex are also shown.

**Table 3:** **Mean values and the correlations of salivary oxytocin (OT) levels and each gaze fixation parameter in children with autism spectrum disorder (ASD)**.

		**Total (***n* = 15**)**		**Male (***n* = 12**)**		**Female (***n* = 3**)**		***t***	**Correlation**		**Partial correlation**
		**Mean**	***SD***	**Mean**	***SD***	**Mean**	***SD***		**Age**	**OT level**	**OT level**
Oxytocin levels (pg/ml)		39.33	23.52	40.3	25.02	35.7	20.11	0.29	−0.171	–	–
Face	%Eyes	36.73	10.12	36.5	9.19	37.7	15.82	0.17	−0.274	**0.461^#^**	0.439
	%Mouth	30	12.61	28.2	11.90	37.3	15.28	1.14	−0.123	−0.215	−0.217
	%Out of AOI	16.33	7.86	17.6	8.04	11.3	5.51	1.26	0.292	−0.013	0.004
People and geometry	%People	36.2	17.76	**31.4**	15.89	**55.3**	11.50	2.42^*^	−0.529^*^	0.268	0.332
	%Geometry	35.4	16.96	**40.0**	15.73	**17.0**	4.36	2.44^*^	0.414	−0.271	−0.342
	%Out of AOI	20.07	7.71	19.6	8.11	22.0	6.93	0.47	0.324	0.115	0.216
Biological motion	%Upright	55.53	20.16	**50.9**	18.74	**74.0**	16.52	**1.94^#^**	−0.269	0.251	0.304
	%Inverted	27.4	15.4	28.8	15.71	21.7	15.50	0.71	0.028	−0.043	−0.064
	%Out of AOI	11	10.11	12.9	10.47	3.3	1.53	1.54	0.452	−0.386	−0.421
Finger pointing	%Pointed	41.93	13.36	50.9	18.74	74.0	16.52	1.30	0.060	0.065	0.131
	%Non-pointed	11.6	8.54	28.8	15.71	21.7	15.50	0.73	0.229	0.312	0.354
	%Out of AOI	42.53	9.96	12.9	10.47	3.3	1.53	0.35	−0.280	−0.314	−0.412

# p < 0.10;

* *p < 0.05*.

For salivary OT levels and gaze fixation, we first tested whether there was an effect of sex on each parameter. No significant difference was observed for salivary OT levels [*t*_(56)_ = 0.35, *n.s*.], indicating a lack of sex difference among OT levels in both groups.

Regarding the relationship between age, salivary OT levels, and respective gaze fixation parameters, the correlation coefficients for these variables are presented in Table 3. First, no significant correlation was found between age and OT levels, as it was for TD children. For variables of gaze fixation, a significant negative correlation was found only for the fixation % of people moving. This result suggests that aging in children with ASD also decreases attention for people moving as it does in TD children. Finally, for salivary OT levels, a marginally significant correlation was found with the % of fixation on eyes in the facial stimuli, indicating that children with ASD and high OT levels were more attentive to the eye area in terms of facial recognition than children with low OT levels.

### Comparison of visual attention for social signals and OT levels in TD children and those with ASD

To examine the interaction among sex and developmental status for OT levels and the fixation duration for social information, the ratio of the percentage of fixation duration for each category was calculated as the dependent variable: Face, eyes-to-mouth; People and Geometry, people-to-geometry; Biological motion, upright-to-inverted; Finger pointing, pointed-to-non-pointed. Subsequently a Two-Way (2 × 2) ANOVA was conducted with sex (male, female) and development status (patients, controls) as between-subjects factors.

**Figure 3:**
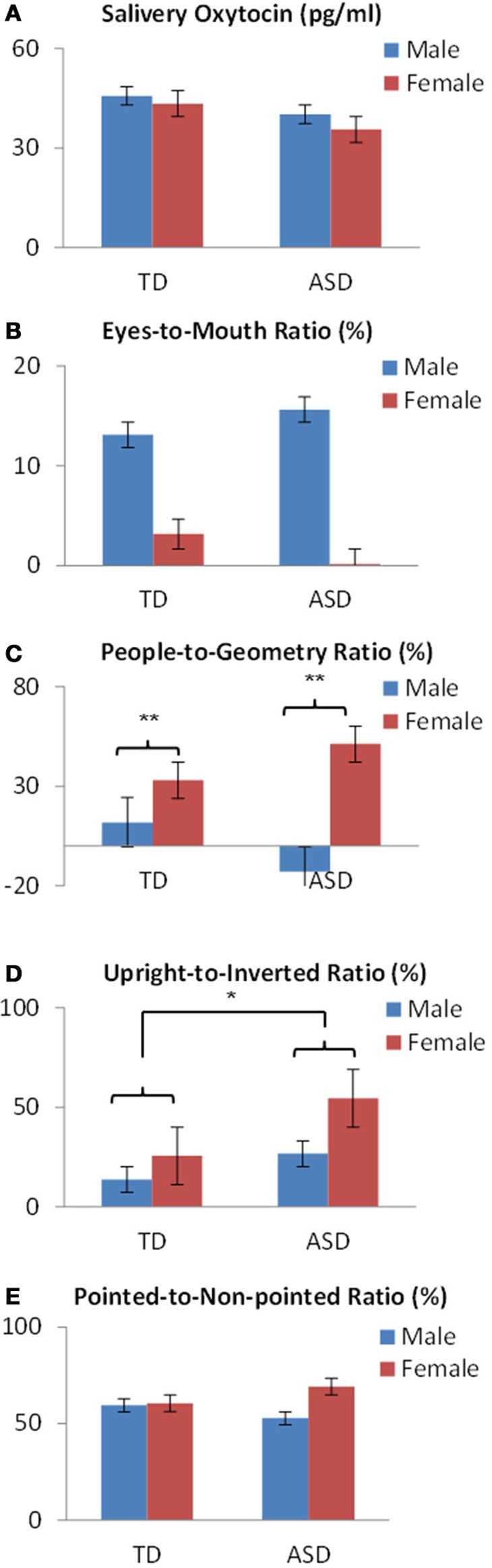
**OT levels and fixation variables by sex and developmental status in preschoolers show (A) no difference in salivary OT levels or (B) in eyes-to mouth ratios**. **(C)** A significant difference of sex was found in people-to-geometry ratios. **(D)** A significant difference of developmental status was found in the upright-to-inverted ratios in bodily motion stimuli. **(E)** No difference was found in the pointed-to-non-pointed ratios of finger pointing stimuli. ^*^p < 0.05, ^**^p < 0.01.

First, for salivary OT levels, there was no main effect of sex (*p* = 0.693) or developmental status (*p* = 0.449), and no sex × developmental status interaction (*p* < 0.897) (Figure 3A).

Next, regarding fixation variables, analysis of the eyes-to-mouth ratio in the face stimuli showed no main effect of either sex (*p* = 0.223) or developmental status (*p* = 0.979), and no sex × developmental status interaction (*p* = 0.790) (Figure 3B). Analysis of the people-to-geometry ratio showed a main effect of sex [*F*_(1,69)_ = 9.73, *p* < 0.01], but no main effect of developmental status (*p* = 0.815) or sex × group interaction (*p* = 0.118), indicating that female children were more attentive to people moving than male children, irrespective of developmental status (Figure 3C). Analysis of upright-to-inverted biological motion showed no main effect of sex (*p* = 0.066), developmental status [*F*_(1, 69)_ = 3.96, *p* < 0.05], or sex × group interaction (*p* = 0.455), indicating that children with ASD were more attentive to biological motion than TDs irrespective of the sex group (Figure 3D). Analysis of pointed-to-non-pointed ratio showed no main effect of sex (*p* = 0.323), development status (*p* = 0.907), or sex × developmental status interaction (*p* = 0.385) (Figure 3E).

## Discussion

This study examined the relationship between gaze fixation for social information and OT levels in preschool children with ASD or typical development. The results revealed a positive association between salivary OT levels and % pointed-at object area in TD children, although no association was found between these variables in children with ASDs. Thus, TD children with high OT levels pay more attention to a pointed-at object than do those with low OT levels. Most gaze fixation variables were dependent on age, with results revealing a positive association of the % of fixation on mouths, geometric patterns, and biological motion. In contrast, a negative association was found between age and the % of fixation on people and pointed-at objects in TD children. Although there was a negative association between age and the fixation duration on people in children with ASD, those tendencies were clearer in TD than in ASD. These results suggest that attention for people moving and pointed-at objects decreases with aging in preschool children, although attention for the mouth, geometric patterns, and biological motion increased with age.

Many previous studies have suggested that OT has a positive effect on attention for social information (Domes et al., 2007a,b; Donaldson and Young, 2008; Guastella et al., 2008; Insel, 2010). Earlier studies also suggested that impairments in social functioning of patients with ASDs were related to dysfunction of the OT mechanism (Andari et al., 2010; Guastella et al., 2010; Kosaka et al., 2012). Moreover, several studies have revealed that patients with ASD have poorer or different visual attention for social information than the amount of visual attention expected to result from typical development (Klin et al., 2002a, 2003). In that context, eye-tracking technology presents numerous advantages for investigating visual social attention in ASD (Guillon et al., 2014). This report is the first study to clarify the association between the pattern of visual attention for social information and OT levels in preschool children with ASD and TD.

We also found a positive association between salivary OT levels and % pointed object areas in the pointing gesture movie for TD children. The ability to follow pointing gestures by others is a significant component in establishing joint attention (Paparella et al., 2011), and various studies have reported a deficit in joint attention as one of the salient signs of ASDs (Warreyn et al., 2007; Redcay et al., 2013). Although there is no direct evidence that OT has a positive effect on joint attention, the idea that social motivation deficits play a central role in ASD has recently gained increasing interest (Chevallier et al., 2012), and it has also been suggested that OT may have a positive effect on enhancing social motivation including joint attention (Stavropoulos and Carver, 2013). In addition, although previous studies have suggested that the mPFC and posterior superior temporal sulcus (pSTS) mediate joint attention (Redcay et al., 2013), recent brain imaging studies have shown that mPFC and pSTS were more activated for social literacy when OT was administered (Gordon et al., 2013). Moreover, several findings have suggested that OXTRs are abundant in the mPFC compared with other brain regions (Skuse and Gallagher, 2009). These findings suggest a positive OT effect for joint attention. Hence, an increase in OT levels may induce enhanced gaze duration at a pointed object.

In the current study, the pattern of visual attention for social information was affected not only by OT levels, but also by age or sex, the development of which represents interactions between these variables. However, results of previous studies have suggested that children with ASD have less attention for social information than TD children; especially for social information variables related to the eyes (Jones and Klin, 2013), people relative to a geometric pattern (Pierce et al., 2011), and biological motion (Klin et al., 2002a,b). In terms of aging effects, our results revealed a positive association with people moving, eye area on the face, and pointed-at objects, and a negative association with geometric patterns in TD preschoolers. These results suggest reduced attention for social information with aging, although this has not received much attention at this point. Furthermore, although this tendency was also observed in children with ASD, the results were not clear because of the insufficient number of samples.

This study confirmed the findings of earlier studies in that female children had more attention for people, whereas male children had more attention for geometric patterns (Pierce et al., 2011). Sex differences reportedly exist for infants' preferences for several properties of the human face and body (Bower, 1989). However, although the present study found that children with ASD were more attentive to biological motion than TD children were, irrespective of sex group, this finding is inconsistent with those of earlier studies (Klin et al., 2002a,b). We were unable to clarify the underlying mechanism, but a possible explanation is the difference of age groups examined in current and previous research. Our participants were preschoolers, whereas most subjects of previous research were infants. As described above, the visual attention for social information can change depending on age. Therefore, aging might be the cause of the discrepancy that we observed. This study also presents some limitations that, if resolved, would improve assessment for ASD. For one, we should analyze the difference between ASD and TD groups using DQ and gender differences as confounding factors. This will be important analyses to conduct since these factors could produce differences in terms of performance and visual attention. Moreover, a larger sample size will be beneficial, therefore, it is necessary to conduct this study again with more participants.

In conclusion, these results demonstrate that OT levels have a positive association with visual attention for finger pointing in TD preschoolers, although no association was found between these variables in children with ASDs. These results suggest that OT is involved in visual attention for social information. Moreover, with the exception of biological motion, age showed a negative relationship with visual attention for social information. However, this study did not directly investigate the neural mechanism underlying this effect. Thus, combining this experimental paradigm with neurophysiological indicators of brain activity (such as imaging techniques) should prove fruitful in further elucidating mechanisms underlying visual attention for social information.

## Funding

This work was supported by a Grant-in-Aid for Scientific Research (B), Young Scientists (B) and Challenging Exploratory Research from the Ministry of Education, Culture, Sports, Science and Technology (MEXT) of Japan (KAKENHI: grant numbers 24300149 and 25560386 to Akemi Tomoda, grant number 25750405 to Takashi X. Fujisawa). Part of this report is the result of “Integrated Research on Neuropsychiatric Disorders” conducted under the Strategic Research Program for Brain Sciences by MEXT of Japan. This work also was partially supported by a research grant from the “Center of Community (COC)” program from the MEXT of Japan to Takashi X. Fujisawa, Daisuke N. Saito, and Akemi Tomoda. Funding organizations had no role in the design or conduct of the study, such as the collection, management, analysis, or interpretation of the data, or the preparation, review, or approval of the manuscript.

### Conflict of interest statement

The authors declare that the research was conducted in the absence of any commercial or financial relationships that could be construed as a potential conflict of interest.
